# Grad student to biotech to pharma: career odyssey of an industrial structural biologist

**DOI:** 10.1063/4.0000867

**Published:** 2025-10-27

**Authors:** David M Dranow

**Affiliations:** UCB, Bainbridge Island, WA, USA

## Abstract

I went to grad school convinced that I wanted to become a professor and run my own lab. However, I ultimately learned that academia wasn't for me, so I left my PhD program with a Master's degree and found a 'temporary' position at a small biotech. This job grew into a career spanning 15 years. During my time in industry I’ve worked for a contract research organization that rebranded twice before being acquired by a mid-size pharma. Throughout my career I’ve learned new structural biology and biophysical techniques, been trained to be a leader, and grown to be the structural biology subject matter expert for early-stage therapeutic projects. I’ll share what that journey was like for me and how it continues to shape how I view the role of structural biology.

